# *In vitro* comparison between α-tocopheryl acetate and α-tocopheryl phosphate against bacteria responsible of prosthetic and joint infections

**DOI:** 10.1371/journal.pone.0182323

**Published:** 2017-07-31

**Authors:** Alessandro Bidossi, Monica Bortolin, Marco Toscano, Elena De Vecchi, Carlo L. Romanò, Roberto Mattina, Lorenzo Drago

**Affiliations:** 1 Laboratory of Clinical Chemistry and Microbiology, IRCCS Galeazzi Orthopaedic Institute, Milan, Italy; 2 Laboratory of Clinical Microbiology, Department of Biomedical Sciences for Health, University of Milan, Milan, Italy; 3 Department of Bone and Joint Infections and Reconstructive Surgery, IRCCS Galeazzi Orthopaedic Institute, Milan, Italy; 4 Department of Public Health, Microbiology and Virology, University of Milan, Milan, Italy; Harvard Medical School, UNITED STATES

## Abstract

Biofilm-related infections represent a recurrent problem in the orthopaedic setting. In recent years, great interest was directed towards the identification of novel molecules capable to interfere with pathogens adhesion and biofilm formation on implant surfaces. In this study, two stable forms of α-tocopherol, the hydrophobic acetate ester and the water-soluble phosphate ester, were tested *in vitro* as coating for titanium prosthesis. Antimicrobial activity against microorganisms responsible of prosthetic and joints infections was assessed by broth microdilution method. In addition, α-tocopherol esters were evaluated for both their ability to hamper bacterial adhesion to and biofilm formation on sandblasted titanium surfaces. Results showed that only α-tocopheryl phosphate displayed antimicrobial activity against the tested strains. Both esters were able to significantly interfere with bacterial adhesion and to prevent biofilm formation, especially by *Staphylococcus aureus* and *Staphylococcus epidermidis*. The activity of α-tocopheryl phosphate was greater than that of α-tocopheryl acetate. Alterations at membrane levels have been reported in literature and may be likely responsible for the interference on bacterial adhesion and biofilm formation shown by α-tocopherol esters. Although further studies are needed to better investigate the mechanisms of action and the spectrum of activity of α-tocopherol esters, these characteristics together with the positive effect on wound healing and immune response, make these molecules promising candidate for coating in order to prevent implant-associated infections.

## Introduction

The term Vitamin E refers to a group of tocopherols and tocotrienols, of which α-tocopherol (α-T) has the highest biological activity [1]. It is commonly known as a powerful fat-soluble antioxidant which is known for its ability to reduce oxidative stress by quenching free radicals formed in the lipid phase of membranes or lipoproteins, affecting the development of cardiovascular diseases, cancers and neurological diseases [2] and positively influencing wound healing [3]. Furthermore, a great interest is directed towards its possible role in gene regulation and cellular signalling [3,4].

α-tocopheryl acetate (vitamin E acetate, α-T-Ac) is the acetic ester of α-T, a viscous, highly hydrophobic oil with a better stability than the unreduced form. This form is commonly used to treat atopic dermatitis and other skin diseases, where the acetate group has been found to be hydrolyzed, thus regaining the redox activity [5].

Recently, the use of Vitamin E on implant surfaces has been proposed to reduce the extent of microbial adhesion by changing the surface of the substratum and by affecting microbial adhesion process. In an *in vitro* experiment, α-T-Ac embedded in poly-lactic acid has been demonstrated to alter bacterial adhesiveness and biofilm formation similarly to the non-ester form α-T [6].

In addition, polyethylene embedded Vitamin E has been shown to reduce adhesion of biofilm-producing *Staphylococcus epidermidis*, *Staphylococcus aureus*, *Escherichia coli*, and *Candida albicans* isolated from biomaterial-associated infections [7–10], but intraspecies differences [10] and contrasting results have been reported [11]. A previous study has also demonstrated that Vitamin E causes a significant reduction of hydrophobicity of *E*. *coli*, being able to prevent the adhesion of this microorganism to nitrocellulose [12].

Bacterial adhesion to implant surface occurring over time during surgical implantation is determined by specific and non-specific interactions between microorganisms and material surface. The adhesion process occurring in the first hours and subsequent biofilm formation on implant surfaces mainly depends on the physicochemical interactions between substratum and microorganisms and are strongly influenced by the physical properties of the surface (roughness, hydrophobicity, electrostatic charge, coating) [13]. Therefore, great efforts are being made to dig out new and reliable strategies to avoid pathogen attachment to implants.

In recent years, a water-soluble form of α-T, α-tocopheryl phosphate (vitamin E phosphate, α-T-P), has been detected in low amounts in human and animal tissues and plasma [14]. Since the hydroxyl group responsible for the antioxidant activity is phosphorylated in α-T-P, this molecule should not have an antioxidant activity *per se*. Rezk *et al*. has proposed that this molecule acts as a pro-vitamin, still maintaining a very strong antioxidant activity and exhibiting some novel regulatory activities in the cells [15,16]. To our best knowledge, no studies have been carried out to assess the antibacterial activity of α-T-P so far.

The present work aimed to evaluate the *in vitro* antimicrobial activity and efficacy in preventing adhesion and biofilm formation of α-T-Ac and α-T-P against microorganisms responsible for prosthetic and joints infections.

## Materials and methods

### Reagents

The following reagents were used: α-tocopheryl acetate (Alfa Aesar, Heysham, UK) and α-tocopheryl phosphate (α-tocopherol phosphate disodium salt; Sigma-Aldrich, Milan, Italy). Stock solutions were prepared by dissolving α-T-Ac in ethanol (500 mg/mL) and α-T-P in sterile distilled water (250 mg/mL).

### Bacterial strains

Clinically relevant strains isolated from patients with prosthetic joint infections at the Laboratory of IRCCS Galeazzi Orthopaedic Institute were used in this study. Five strains each of *S*. *epidermidis*, *S*. *aureus*, *Pseudomonas aeruginosa* and *Propionibacterium acnes* were included in the study. Strain identification was performed by using the Vitek^®^ 2 Compact system (bioMérieux, Marcy l'Etoile, France), which settles colorimetric reagent cards that are incubated and interpreted automatically. The cards have 64 wells each containing an individual test substrate for measuring various metabolic activities (e.g. acidification, alkalization, enzyme hydrolysis, and growth in the presence of inhibitory substances). In particular, Vitek^®^ GN ID card, Vitek^®^GP ID card, and Vitek^®^ ANC ID card were used for microbial identification while antimicrobial susceptibility testing of staphylococci and *P*. *aeruginosa* was carried out on Vitek^®^ AST 632 and 202 cards, respectively.

Stain identification was further confirmed through DNA sequencing of about 80 bp of variable regions V1 and V3 of the 16S rRNA gene by Pyrosequencing (PSQ96RA, Diatech, Jesi, Italy) as previously reported. Obtained sequences were pasted in BLAST to perform identification (http://blast.ncbi.nlm.nih.gov/Blast.cgi).

Strains were stored at −80°C in proper broths enriched with 10% glycerol (VWR Chemicals, Leuven, Belgium) until testing.

### Antimicrobial activity by broth microdilution

The bacteriostatic and bactericidal activities of α-T-Ac and α-T-P were evaluated against the above-mentioned bacterial strains by assessing the Minimum Inhibitory Concentration (MIC) and the Minimum Bactericidal Concentration (MBC). The MIC, defined as the lowest concentration able to inhibit bacterial growth, was determined by broth microdilution method, in accordance with EUCAST guidelines [17] except for the use of Brain Heart Infusion broth (BHI; Biomérieux, Marci l’Etoile, France) instead of Mueller Hinton broth. Briefly, for each strain a suspension with a density equal to 0.5 McFarland (1.5×10^8^ CFU/mL) was prepared in BHI. To support growth of *P*. *acnes*, broth was supplemented with 5% defibrinated sheep blood (Liofilchem, Roseto degli Abruzzi, Italy). After proper dilutions, an aliquot from each suspension (10^5^ CFU/mL) was inoculated into a 96-wells microtiter plate containing 2-fold serial dilutions of α-T-Ac and α-T-P. Due to solubility limits, the maximum concentration used for both formulations was 200 mg/mL. Growth controls were performed by inoculating bacterial suspensions in BHI alone. MIC values were read after 24 h of incubation at proper conditions (except for *P*. *acnes* that was incubated for 48 h). The MBC was determined by plating 10 μL from each well showing no turbidity onto Tryptic Soy Agar plates (TSA; Merck, Darmstadt, Germany) or Schaedler Blood Agar plates (SCH; bioMérieux, Marcy l'Etoile, France) in the case of P. acnes. TSA was incubated in aerobiosis at 37°C for 24 hours, while SCH was incubated at 37° for 48 hours in anaerobiosis. The anaerobic atmosphere was created in jars by anaerobic gas generating sachets (OxoidTM AnaeroGenTM 2.5L, Thermo Scientific). After incubation at proper conditions, MBC was read as the lowest concentration able to kill 99.9% of the initial inoculum.

### Anti-adhesion properties on sandblasted titanium

The anti-adhesion activity of vitamin E was evaluated against one representative strain for each species. *P*. *acnes* was not included in these experiments because none of the tested isolates was able to adhere or to form biofilm in our *in vitro* setting. Sterile sandblasted titanium discs with a diameter of 25 mm and a thickness of 5 mm (Adler Ortho, Cormano, Italy) were used as substrate for bacterial adhesion. Discs were coated by spreading uniformly the proper amount of α-T-Ac or α-T-P on the upper disc surface in order to achieve a final concentration of 5 mg/cm2. Uncoated discs were used as controls. Subsequently, a bacterial overnight culture was resuspended in BHI at a density of 1.0 × 10^7^ CFU/mL, and 400 μL of microbial suspension was inoculated onto coated and uncoated discs. The test was performed in triplicate for each strain. After 30, 60 and 120 min of incubation, discs were rinsed twice by submersion in 5 mL of sterile saline to remove non-adherent bacteria. Then, discs were immersed in 5 ml of a solution of 0.1% w/v dithiothreitol (DTT; Sigma-Aldrich, Milan, Italy) and mechanically stirred for 15 min at room temperature to detach bacteria adhered to the discs [18]. Proper dilutions of the obtained fluids were plated onto TSA and incubated at 37°C in aerobic atmosphere for 24 h for CFU count.

### Prevention of biofilm formation on sandblasted titanium

The ability of α-T-Ac or α-T-P to prevent biofilm development was assessed after chemical detachment of bacterial cells followed by conventional plate counting (CFU method). To evaluate the barrier effect of the two compounds, titanium discs were coated with 5 mg/cm^2^ of α-T-Ac or α-T-P, as described above, and then used as substrate for biofilm formation. Uncoated discs were used as controls. A bacterial overnight culture was resuspended in BHI at a density of 1.5 × 10^8^ CFU/mL, and 200 μL of microbial suspension was inoculated into sterile 6-wells polystyrene plates containing coated and uncoated discs immersed in 4.8 mL of BHI. Plates were incubated at 37°C in aerobic atmosphere for 24 and 48 h. The test was performed in triplicate for each strain. At the end of incubation, discs were washed with sterile normal saline and then treated with DTT as described above. Cells detached from biofilm were seeded onto TSA and incubated at 37°C in aerobic atmosphere for 24 h for CFU count.

### Statistical analysis

Results were expressed as mean ± standard deviation (SD). Assessment of normality was performed by using Kolmogorov-Smirnov test. Comparisons were carried out by means of two-way ANOVA followed by Tukey’s post-hoc tests. A value of P equal or less than 0.05 was used as threshold for statistical significant differences.

## Results

### Evaluation of the antimicrobial activity

Full MIC and MBC data are displayed in Table 1. Only α-T-P displayed antimicrobial activity against the tested strains, while the acetate form was ineffective at the tested concentrations. *P*. *acnes* seemed to be the most susceptible microorganism to α-T-P, with MIC values ranging from 0.20 to 1.60 mg/mL. MIC values for *S*. *epidermidis* and *S*. *aureus* were more variable (3.20–100 mg/mL and 25–100 mg/mL, respectively). *P*. *aeruginosa* was the least susceptible species among the tested microorganisms, with MIC values varying from 100 to 200 mg/mL α-T-P was also found to be bactericidal against *P*. *acnes*, *S*. *epidermidis* and *S*. *aureus*, but not against *P*. *aeruginosa*. Excluding *P*. *aeruginosa*, 53% of the isolates showed MBCs equal to MICs, while the rest were characterized by MBCs 2–8 times higher than the corresponding MIC.

**Table 1:** MIC and MBC values of α-tocopheryl phosphate and α-tocopheryl acetate.

	α-tocopheryl phosphate		α-tocopheryl acetate		Resistance to conventional antibiotics
	MIC	MBC	MIC	MBC	
*S*. *aureus*					
Strain 1	25	25	>200	>200	-
Strain 2	25	25	>200	>200	-
Strain 3	100	100	>200	>200	Methicillin
Strain 4	100	100	>200	>200	Methicillin
Strain 5	100	200	>200	>200	Methicillin, teicoplanin (MIC = 4)
*S*. *epidermidis*					
Strain 1	3.20	25	>200	>200	Methicillin
Strain 2	25	100	>200	>200	Methicillin
Strain 3	50	100	>200	>200	Methicillin
Strain 4	100	100	>200	>200	Methicillin
Strain 5	100	100	>200	>200	Methicillin
*P*. *aeruginosa*					
Strain 1	100	> 200	>200	>200	Ceftazidime (MIC = 16), Ciprofloxacin (MIC ≥ 4)
Strain 2	200	> 200	>200	>200	-
Strain 3	200	> 200	>200	>200	Ceftazidime (MIC ≥ 64), Ciprofloxacin (MIC ≥ 4)
Strain 4	200	> 200	>200	>200	Ceftazidime (MIC = 16), Ciprofloxacin (MIC ≥ 4)
Strain 5	200	> 200	>200	>200	-
*P*. *acnes*					
Strain 1	0.20	0.20	>200	>200	-
Strain 2	0.20	0.20	>200	>200	-
Strain 3	0.20	0.80	>200	>200	-
Strain 4	0.40	0.80	>200	>200	-
Strain 5	1.60	3.20	>200	>200	-

Values are expressed as mg/mL.

### Evaluation of the anti-adhesive activity

**Fig 1:**
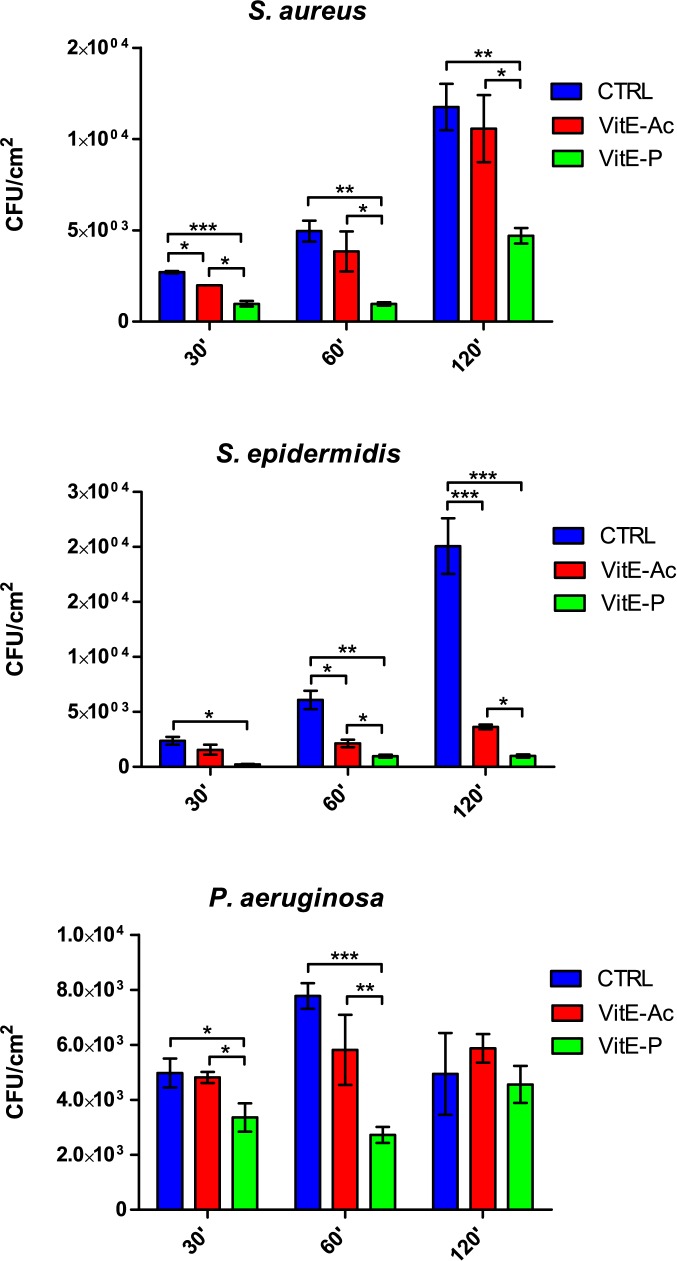
Anti-adhesive activity of α-tocopheryl acetate and α-tocopheryl phosphate. Blue bars = control; red bars = tocopheryl acetate (5 mg/cm^2^); green bars = α-tocopheryl phosphate (5 mg/cm^2^). Results are expressed as mean ± SD. *P < 0.05, **P < 0.01, ***P < 0.001.

The results of the anti-adhesive activity of the two tested compounds are shown in Fig 1.

α-T-Ac caused a significant decrease in adhesion of *S*. *epidermidis* at 60 min and 120 min. *S*. *aureus* adhesion was significantly decreased only after 30 min, while ability of *P*. *aeruginosa* to adhere to titanium discs was not affected by treatment with α-T-Ac.

For all the tested strains, α-T-P elicited a higher decrease in the amount of adhered cells than α-T-Ac. Major effects were seen against *S*. *epidermidis*, where adhesion was almost totally inhibited (84–95% respect to the control), and *S*. *aureus* (60–78%). In the case of *P*. *aeruginosa*, α -T-P was able to reduce adhesion at the earlier timepoints (7–65% reduction).

When comparing the two esters, the phosphate form was significantly more effective than the acetate one in reducing adhesion of *S*. *aureus* at all time points. Significant differences between the two formulations were also observed for *S*. *epidermidis* at 60 and 120 min and *P*. *aeruginosa* at 30 and 60 min.

### Prevention of biofilm formation

**Fig 2:**
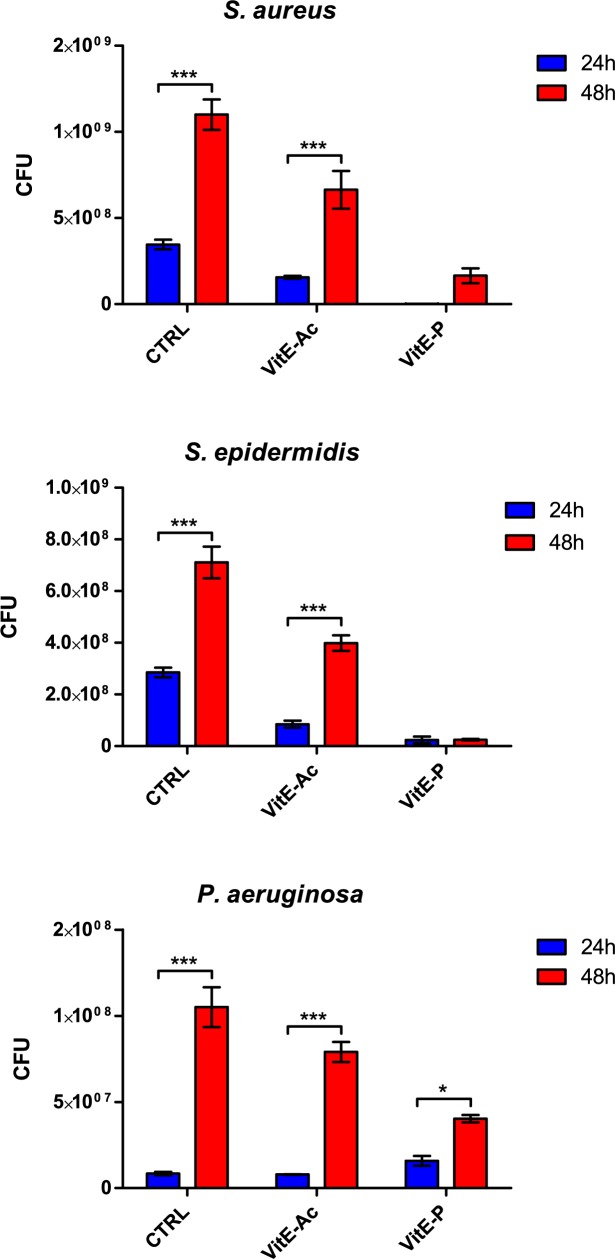
Prevention of biofilm formation by α-tocopheryl acetate and α-tocopheryl phosphate. Blue bars = control; red bars = tocopheryl acetate (5 mg/cm^2^); green bars = α-tocopheryl phosphate (5 mg/cm^2^). Results are expressed as mean ± SD. *P < 0.05, **P < 0.01, ***P < 0.001, ****P < 0.0001.

Both α-T-Ac and α-T-P showed an inhibitory effect on the development of biofilm on titanium discs by *S*. *epidermidis* and *S*. *aureus* (Fig 2). In particular, both esters caused a significant reduction of biofilm formation by *S*. *epidermidis*, which was statistically significant at 24 and 48 h: 92–97% for α-T-P and 44–70% for α-T-Ac. Formation of biofilm by *S*. *aureus* was inhibited at 24 h by both formulations (100% reduction versus control for α-T-P, 55% for α-T-Ac), but only by α-T-P at 48 h (85% reduction). In the case of *P*. *aeruginosa*, no effect was seen at the earlier timepoint, while biofilm formation was significantly reduced after 48 h by phosphate ester only (49%).

When the inhibitory activity on biofilm formation by the two compounds was compared, a significant difference was observed at 48 h for all the tested strains, with the phosphate ester being the more efficient.

## Discussion

Implant-related infections represent a recurrent problem after surgeries. The fate of implanted biomaterials can be seen as a “race for the surface” between tissue integration and biofilm formation [19]. The initial phases of biofilm development on biomedical devices are the most critical, as once a biofilm has established on the surface it is difficult to eradicate, because the bacteria residing in the biofilm are protected from the host immune system and from antibiotics. Preventive measures should aim primarily at discouraging biofilm formation by finishing implant material surfaces with repellent coatings. Hence, in recent years, great interest was directed to the identification of new molecules and compounds, compatible with the human body and the implant materials, capable to interfere with pathogens attachment to surfaces [20].

Vitamin E is a group of structurally related compounds with beneficial biological activity on animals and humans. α-T is the predominant form of vitamin E in most human and animal tissues, including plasma [21,22], and has the highest vitamin E activity for animals and humans [1].

Here we tested two stable forms of α-T, the viscous and highly hydrophobic acetate ester and the water-soluble phosphate ester, as coatings for titanium prosthesis. To our knowledge, this is the first study evaluating the antimicrobial activity of α-T-P. In this work, α-T-P showed a good anti-microbial activity against all the tested stains, although at variable concentrations depending on the microbial species. In our microbiological settings, this antimicrobial activity was always higher compared to that of α-T-Ac, so that it may be hypothesized that the high hydrophobicity of α-T-Ac could limit its solubility into the growth medium.

In this work, α-T-P and α-T-Ac were evaluated for their ability to either hamper bacterial adhesion and biofilm formation by microorganisms responsible of prosthetic and joints infections such as *S*. *aureus*, *S*. *epidermidis* and *P*. *aeruginosa*. *P*. *acnes* strains were necessarily excluded from these evaluations because strains from our collection showed a low efficiency to adhere and form biofilm. The amount of Vitamin on the surface of titanium discs was chosen in order to ideally obtain sub-inhibitory concentration for all the tested bacteria.

Bacterial adhesion to substrate represents the first crucial step towards infection and usually occurs during the first hours after invasion of the host tissues. The two vitamin E esters, in particular the phosphate one, have demonstrated a notable anti-adhesive ability against the tested strains of *S*. *epidermidis* and *S*. *aureus*. By contrast, ability of *P*. *aeruginosa* to adhere to titanium discs was only partially affected by α-T-P, whereas α-T-Ac was ineffective.

Bacterial adhesion is generally followed by production of biofilm to establish infection. For this reason, we evaluated whether bacteria were able to develop a mature biofilm on vitamin-treated substrates. The amount of biofilm produced on coated and uncoated discs was assessed by CFU count method, as described elsewhere [23,24]. Such method allows an indirect assessment of biofilm by the estimation of the number of viable cells embedded into biofilm. For evaluating biofilm matrix we could not adopt the traditional colorimetric assay [25], because α-T-P coated on titanium substrates strongly binds crystal violet, thus interfering with spectrophotometric readings.

Both α-T-Ac and α-T-P coatings showed an inhibitory effect against the development of biofilm on titanium discs by *S*. *epidermidis* and *S*. *aureus*. As observed in the adhesion assay, also in this case the activity of α-T-P was greater than that observed for α-T-Ac. Interestingly, α-T-P caused a reduction in amount of staphylococci embedded in biofilm of more than 90%, thus it may be hypothesized that the rate of persister/tolerant cells could be very low after treatment and subsequent production of biofilm by these cells heavily affected. To evaluate if anti-adhesive and anti-biofilm effects were exerted directly on the surface of titanium discs or due to the vitamin E dissolved in the medium, we measured α-T-P and α-T-Ac concentrations in the broth by means of high-performance liquid chromatography (HPLC), in all the tested conditions (S1 Table). Both α-tocopherol forms were found in very low amounts in the medium with no significant differences between the two vitamin E forms, meaning that both the formulations remained steadily absorbed to the substrate and did not diffuse into the medium.

Our data confirmed findings of previous works evaluating anti-adhesive properties of α-T and α-T-Ac containing biomaterials [6,8,10], even if contrasting results and relevant intraspecies differences were observed in these studies.

Even though Vitamin E is mainly known for its antioxidant properties and for limiting ROS damage of healing tissue, many studies focused on its immunomodulatory capabilities and stimulation of cell proliferation influencing transcriptional activity and cell signaling.

Although contrasting results have been reported [3], α-T has been shown to be able to stimulate immune cells chemotaxis [26, 27], phagocytosis [28], T-cell-mediated immune functions and macrophage activity [29,30], to modulate cytokines release [31,32], expression of connective tissue growth factor (CTGF) [33] and activation of fibroblasts [34]. Recently, α-T-P has proved to modulate cellular activities similarly or better than α-T [35] and new roles such as the induction of VEGF expression and an increasing in vitro angiogenesis have been also observed [16].

For these reasons and because it was found to potentiate the antimicrobial activity of tigecycline and daptomycin against MRSA when tested *in vivo* in mice, α-T was speculated to act as an immune enhancer possibly by modulating NK cells and leukocytes [31,36]. In the present study, we observed that α-T-P displays a higher antimicrobial activity than α-T-Ac and α-T in the literature. The observed differences between the tocopherols forms may be likely due to the enhanced solubility of the phosphate form compared to α-T, which makes α-T-P more suitable to be tested in classic *in vitro* microdilution assay. Indeed, Andrade *et al*. were unable to determine the MIC of α-T by performing the same microdilution assay. However, the combination of α-T with aminoglycosides showed a decrease in MIC in respect to the antibiotic alone [37]. The authors suggested that α-T is able to provoke perturbations in the bacterial membrane, altering its fluidity and making it more susceptible to penetration by various substances, particularly antibiotics. This thesis is supported by the work of Uberos *et al*., where the presence of α-T-P caused a reduction of hydrophobicity of the surface of *E*. *coli*, impacting on the capacity to adhere to nitrocellulose [12]. These alterations at membrane levels may be likely responsible for the interference shown by α-T-P on bacterial adhesion and biofilm formation found in our study.

## Conclusions

α-T-P and α-T-Ac are able to significantly interfere with bacterial adhesion and to prevent biofilm formation, especially by *S*. *aureus* and *S*. *epidermidis*, generally considered the most frequent pathogens responsible of prosthetic joint infections. In our experiments, only α-T-P has been seen to have a direct antimicrobial effect. Although further studies are needed to better investigate the mechanisms and the spectrum of activity of α-T-P and α-T-Ac, these characteristics, taken together with the positive effect on wound healing and immune response, could make these molecules promising candidate for coatings in order to prevent implant-associated infections and even ameliorate the post-operative course.

## Supporting information

S1 Table Concentration of α-tocopheryl phosphate and α-tocopheryl acetate dissolved in the medium. α-T-P = α-tocopheryl phosphate; α-T-Ac = α-tocopheryl acetate; N.D. = not detectable.
(DOC)
