# Supplementary material for: In vitro comparison between α-tocopheryl acetate and α-tocopheryl phosphate against bacteria responsible of prosthetic and joint infections
Source: PLoS One. 2017 Jul 31;12(7):e0182323. doi: 10.1371/journal.pone.0182323 (PMC5536291; doi:10.1371/journal.pone.0182323)
Supplement: S1 Table — α-T-P = α-tocopheryl phosphate; α-T-Ac = α-tocopheryl acetate; N.D. = not detectable. (DOC) [file pone.0182323.s001.doc]

**S1 Table. Concentration of α-tocopheryl phosphate and α-tocopheryl acetate dissolved in the medium.**

|  | **(µg/mL)** |
| --- | --- |
| α-T-P 30 min | 0.209 |
| α-T-P 60 min | 0.682 |
| α-T-P 120 min | 0.330 |
| α-T-P 24 h | 0.220 |
| α-T-P 48 h | 0.473 |
| α-T-Ac 30 min | N.D. |
| α-T-Ac 60 min | 0.297 |
| α-T-Ac 120 min | 0.264 |
| α-T-Ac 24 h | N.D. |
| α-T-Ac 48 h | 0.121 |

α-T-P = α-tocopheryl phosphate

α-T-Ac = α-tocopheryl acetate

N.D. = not detectable
